# A Receptor Guanylate Cyclase, Gyc76C, Mediates Humoral, and Cellular Responses in Distinct Ways in *Drosophila* Immunity

**DOI:** 10.3389/fimmu.2020.00035

**Published:** 2020-01-28

**Authors:** Shinzo Iwashita, Hiroaki Suzuki, Akira Goto, Tomohito Oyama, Hirotaka Kanoh, Takayuki Kuraishi, Naoyuki Fuse, Tamaki Yano, Yoshiteru Oshima, Julian A. T. Dow, Shireen-Anne Davies, Shoichiro Kurata

**Affiliations:** ^1^Graduate School of Pharmaceutical Sciences, Tohoku University, Sendai, Japan; ^2^Graduate School of Life Sciences, Tohoku University, Sendai, Japan; ^3^PRESTO, Japan Science and Technology Agency, Tokyo, Japan; ^4^Institute of Molecular, Cell and Systems Biology, College of Medical, Veterinary and Life Sciences, University of Glasgow, Glasgow, United Kingdom

**Keywords:** receptor-type guanylate cyclase, humoral immune responses, cellular immune responses, *Drosophila*, innate immunity

## Abstract

Innate immunity is an evolutionarily conserved host defense system against infections. The fruit fly *Drosophila* relies solely on innate immunity for infection defense, and the conservation of innate immunity makes *Drosophila* an ideal model for understanding the principles of innate immunity, which comprises both humoral and cellular responses. The mechanisms underlying the coordination of humoral and cellular responses, however, has remained unclear. Previously, we identified Gyc76C, a receptor-type guanylate cyclase that produces cyclic guanosine monophosphate (cGMP), as an immune receptor in *Drosophila*. Gyc76C mediates the induction of antimicrobial peptides for humoral responses by a novel cGMP pathway including a membrane-localized cGMP-dependent protein kinase, DG2, through downstream components of the Toll receptor such as dMyD88. Here we show that Gyc76C is also required for the proliferation of blood cells (hemocytes) for cellular responses to bacterial infections. In contrast to Gyc76C-dependent antimicrobial peptide induction, Gyc76C-dependent hemocyte proliferation is meditated by a small GTPase, Ras85D, and not by DG2 or dMyD88, indicating that Gyc76C mediates the cellular and humoral immune responses in distinct ways.

## Introduction

The innate immune system is a powerful and evolutionarily well-conserved barrier to infectious pathogens (1, 2). The fruit fly *Drosophila melanogaster* is an excellent model organism for deciphering the basic principles of innate immunity, which comprises both humoral and cellular responses (3–5). Induction of antimicrobial peptides (AMPs) in the fat body, the functional equivalent of the mammalian liver, is a humoral response in *Drosophila* controlled by two distinct innate immune signaling pathways, the Toll and immune deficiency (imd) pathways (4, 6). Studies of the Toll receptor, which is involved in host-defense in *Drosophila*, led to the discovery of a Toll-like receptor regulating innate immunity in mammals (1, 2, 7, 8). The Toll and imd pathways are mechanistically similar to the mammalian nuclear factor-kappa B signaling pathways, the Toll-like receptor/interleukin-1 receptor signaling pathway and the tumor necrosis factor-α receptor signaling pathway, respectively (2). Both pathways are mediated by several factors, including the Toll receptor and *Drosophila* myeloid differentiation primary response 88 (dMyD88) adaptor protein, which mediates the Toll pathway; and peptidoglycan recognition protein-LE and peptidoglycan recognition protein-LC receptors, and Relish transcriptional factor, which mediate the imd pathway (4, 6). The Toll pathway is mainly involved in immune defense against fungal and Gram-positive bacterial infections, whereas the imd pathway is mainly involved in immune defense against Gram-negative bacterial infections (3, 6). Upstream of the Toll receptor, peptidoglycan recognition protein-SA and Gram-negative bacteria-binding protein-1 are involved in the recognition of Gram-positive bacteria and Gram-negative bacteria-binding protein-3 is involved in the recognition of fungi. These recognition proteins activate modular serine protease (ModSP), which activates the serine protease cascade (9–12). The Spätzle-processing enzyme is then activated to cleave the cytokine-like protein Spätzle (Spz). Processed Spz binds to the Toll receptor to activate the Toll pathway.

Cellular responses in *Drosophila* are primarily carried out by the blood cells (hemocytes), and include phagocytosis, hemocyte proliferation, and encapsulation by differentiated hemocytes called lamellocytes (3, 13) Recent reports demonstrated crucial roles for hemocytes in host defense against various bacterial infections (14–16), and identified the involvement of several key factors in the phagocytosis of different pathogens, hemocyte proliferation, hemocyte differentiation, and parasite encapsulation (17–20). Two waves of hematopoiesis occur during *Drosophila* development. The first population of hemocytes derives from the head mesoderm in the embryo producing two main classes of hemocytes called plasmatocytes and crystal cells (21–25). The second hematopoiesis occurs during the larval stage in a specialized organ called the lymph gland (26). Lymph glands are responsible for producing larval hemocytes comprising ~90% of plasmatocytes, ~5% of crystal cells, and a third class of cells named lamellocytes, which are generated upon infection by parasitic wasps (26–28). A number of previous studies have demonstrated the involvement of these hemocytes during infection, but relatively little is known about the control and coordination of humoral and cellular immune responses for eliminating invaders.

We previously identified genes capable of activating immune responses by establishing a genome-wide gain-of-function genetic screen based on modular misexpression using GAL4/UAS in *Drosophila* (29, 30). Use of this screening system led to the identification of a receptor-type guanylate cyclase (rGC), Gyc76C, which produces cyclic guanosine monophosphate (cGMP) and mediates AMP induction of humoral responses through the downstream Toll-receptor components dMyd88, Pelle, Tube, and Dif/Dorsal (nuclear factor-kappa B) in parallel with the Toll receptor (Kanoh et al., under revision). This Gyc76C-induced cGMP signaling pathway is mediated by the membrane-localized cGMP-dependent protein kinase (cGK) DG2, encoded by the gene *dg2* (*foraging*) and by protein phosphatase 2A, which is crucial for host survival against Gram-positive bacterial infections in *Drosophila* (Kanoh et al., under revision). Here we report that Gyc76C is also required for hemocyte proliferation in response to bacterial infections. In contrast to Gyc76C-dependent AMP induction, however, Gyc76C-dependent hemocyte proliferation is meditated by a small GTPase, Ras85D, and not by DG2 or dMyD88, indicating that the Gyc76C-mediated cellular response and the Gyc76C-mediated humoral response are differentially regulated. These findings indicate that Gyc76C is an immune receptor that differentially mediates both cellular and humoral immune responses.

## Materials and Methods

### Fly Stocks Used in the Study

Fly stocks used in the study are summarized in Table 1.

**Table 1:** Fly stocks used in this study.

**Stock name**	**Genotype**	**Donator**	**Reference**
UAS-dg2-RNAi	P{KK101298}VIE-260B	VDRC	
UAS-Ras85D-RNAi	w[1118]; P{GD12553}v28129	VDRC	
c564-GAL4	w[1118]; P{w[+mW.hs]=GawB} c564	Dr. Perrimon	
Cg-GAL4	w[1118]; P{w[+mC]=Cg-GAL4.A}2	Bloomington Stock Center	
srpD-GAL4	w[1118]; P{srp-GAL4}	Dr. Meister	PLoS Biol 2004; 2:E196.
Ras85D^EY00505^	y[1] w[67c23]; P{w[+mC]y[+mDint2]=EPgy2} Ras85D[EY00505]	Bloomington Stock Center	
spz^rm7^	ru[1] th[1] st[1] kni[ri-1] rn[roe-1] p[p] e[1] spz[4]/TM3	Dr. Anderson	Cell 1994; 76:677–88.
Relish^E20^	w[1118]; RelE20, ebony(+)	Drs. Hultmark and Reichhart	Mol Cell 1999; 4:827–37.
dMyD88^kra1^	w; dMyD88[kra1]	Dr. Imler	Mech Dev 2003; 120:219–26.
UAS-Gyc76C	w[1118]; P{w[+mC]=UAS-Gyc76C.MYC}1/CyO, P{w[+mC]=act-lacZ.B}CB1	Dr. Kolodkin	J Neurosci 2004; 24:6639–49.
gyc76C^KG03723^	y[1] w[67c23]; P{y[+mDint2] w[BR.E.BR]=SUPor-P}Gyc76C[KG03723] ry[506]	Dr. Kolodkin	J Neurosci 2004; 24:6639–49.
UAS-Gyc76C^D945A^	w[1118]; P{w[+mC]=UAS-Gyc76C.D945A}3-1	Dr. Kolodkin	J Neurosci 2004; 24:6639–49.
UAS-PDE5/6	w[1118]	Dr. Davies	Biochem J 2006; 393(Pt 2):481–8.
UAS-ModSP		Dr. Lemaitre	Proc Natl Acad Sci USA 2009; 106:12442–7.
UAS-Gyc76C RNAi	w[1118];	Dr. Davies	Peptides 2012; 34:209–18.
hml-GAL4	w[1118]; P{w[+mC]=Hml-GAL4.G}5-6	Dr. Goto	Dev Biol 2003; 264:582–91.

*RNAi, RNA interference; VDRC, Vienna Drosophila Resource Center*.

### Bacterial Infection

The following bacteria were used for infection: *Escherichia coli* (K-12), *Erwinia carotovora carotovora 15* (Ecc15), *Staphylococcus aureus* (ATCC14801, wood46), *S. saprophyticus* (GTC0205), and *Enterococcus faecalis* (IFO12964). The flies were raised on a standard cornmeal-yeast agar medium. Flies were infected with bacterial strains by injecting ~70 nl of a suspension of each bacterial strain per fly at 3–5 days after eclosion. The optical density at 600 nm for each bacterial suspension was as follows: *E. faecalis* (0.0001), *S. saprophyticus* (1.0), *S. aureus* (0.0001), and *Ecc15* (1.0). Survival experiments were performed with 30 flies of each genotype at 28°C. Surviving flies were counted daily by transferring the flies to fresh vials. For larval infection, overnight *S. aureus* and *E. coli* cultures were concentrated by centrifugation. The pellets were washed with phosphate-buffered saline (PBS) and the larvae were then pricked with a fine tungsten needle that had been dipped in a pellet of concentrated bacteria.

### Total RNA Isolation and Real-Time PCR

Total RNAs were isolated from each genotype of ~20 flies or larvae with Trizol reagent (GIBCO/BRL). Total RNA (1 μg) was used for cDNA synthesis with ReverTraAce reverse transcriptase (Toyobo) and oligo(dT) 15 primer (Promega). Using the first-strand cDNA (0.5 μl), real-time polymerase chain reaction (PCR) was performed using a LightCycler (Roche Diagnostics). *Rp49* was used as the internal control. The primers used for real-time PCR were as follows (F = forward, R = reverse):

*Rp49*: AGATCGTGAAGAAGCGCACCAAG (F); CACCAGGAACTTCTTGAATCCGG (R)

*Gyc76C*: AGCTACCCCAACTGGGAGAT (F); TGACTCGAGTGCACTTCACC (R)

*dg2*: ATTACTGGTCGCTGGGAGTG (F); AGAAGCCATCGAACCATTTG (R)

*Drs*: TTGTTCGCCCTCTTCGCTGTCCT (F); GCATCCTTCGCACCAGCACTTCA (R)

*Dpt*: GTTCACCATTGCCGTCGCCTTAC (F); CCAAGTGCTGTCCATATCCTCC (R)

*Def* : TTGAACCCCTTGGCAATGCA (F); AGTTCTTCGTTCTCGTGGCT (R)

*CecA1*: CATCTTCGTTTTCGTCGCTC (F); CGACATTGGCGGCTTGTTGA (R)

*Att*: GTGGTGGGTCAGGTTTTCGC (F); TGTCCGTTGATGTGGGAGTA (R)

*Mtk*: AACTTAATCTTGGAGCGA (F); CGGTCTTGGTTGGTTAG (R)

*Dros*: CCATCGTTTTCCTGCT (F); CTTGAGTCAGGTGATCC (R)

### Colony Forming Unit (CFU) Assay

Flies were collected at 0, 6, 24, and 48 h after injection of each bacterial strain and sterilized with 70% ethanol. A total of 14 flies of each genotype was homogenized in 500 μl of the appropriate bacterial medium, serially diluted, and plated onto the appropriate plates (Luria Bertani medium for *E. faecalis*; nutrient broth medium for *S. aureus* and *S. saprophyticus*).

### Hemocyte Staining

Third instar larvae were dissected in Schneider's *Drosophila* medium containing 14% fetal bovine serum at 6 h after infection. Circulating hemocytes were fixed with methanol/water/acetic acid (95:4:1) for 20 min, permeabilized with cold methanol for 15 min, incubated overnight with anti-PH3 (Cell Signaling Technology) diluted 140-fold in PBT (PBS containing 0.1% Triton-X 140), washed, and incubated with Cy-3 anti-rabbit IgG diluted 500-fold in PBT (Jackson ImmunoResearch). The cells were stained with 4′,6-diamidino-2-phenylindole (DAPI; MilliporeSigma) in PBS to visualize nuclei and observed with a Zeiss Axioplan 2 microscope. To count hemocytes, the hemolymph from 10 third-instar larvae per sample was collected in 50 μl PBS. The hemocyte number was counted using a hemocytometer. We counted at least 10 samples and calculated the number of hemocytes per larva.

### Co-immunoprecipitation Assay

*Drosophila* S2 cells were maintained at 25°C in Schneider's Drosophila medium (Life Technologies) and transfected with V5-tagged Ras85D and a FLAG-tagged wild-type Gyc76C or Gyc76C mutant lacking a kinase homology domain. Cell lysates with lysis buffer (30 mM Tris, pH 7.5, 150 mM NaCl, and 1% CHAPS) were incubated with anti-FLAG M2 monoclonal antibody (MilliporeSigma) for 2 h at 4°C, and then with Dynabeads M280 (Life Technologies) for 2 h at 4°C. After washing with wash buffer (30 mM Tris, pH 7.5, 500 mM NaCl, and 1% CHAPS), the bead-captured proteins were eluted with sodium dodecyl sulfate (SDS) sample buffer (50 mM Tris-HCl, 200 mM β-mercaptoethanol, 2% SDS, 0.0125% bromophenol blue, and 14% glycerol) at 140°C for 5 min. The proteins were separated by 14% SDS-polyacrylamide gel electrophoresis and transferred to polyvinylidene difluoride membranes (Hybound-P, GE Healthcare) and then analyzed with anti-V5-tag monoclonal antibody (MBL Life Science) and anti-FLAG antibody. Blots were visualized with the ECL-Western Blotting Analysis system (GE Healthcare).

## Results

### Expression of Gyc76C in Both the Fat Body and Hemocytes Is Required for Self-Defense Against Gram-positive Bacteria

**Figure 1:**
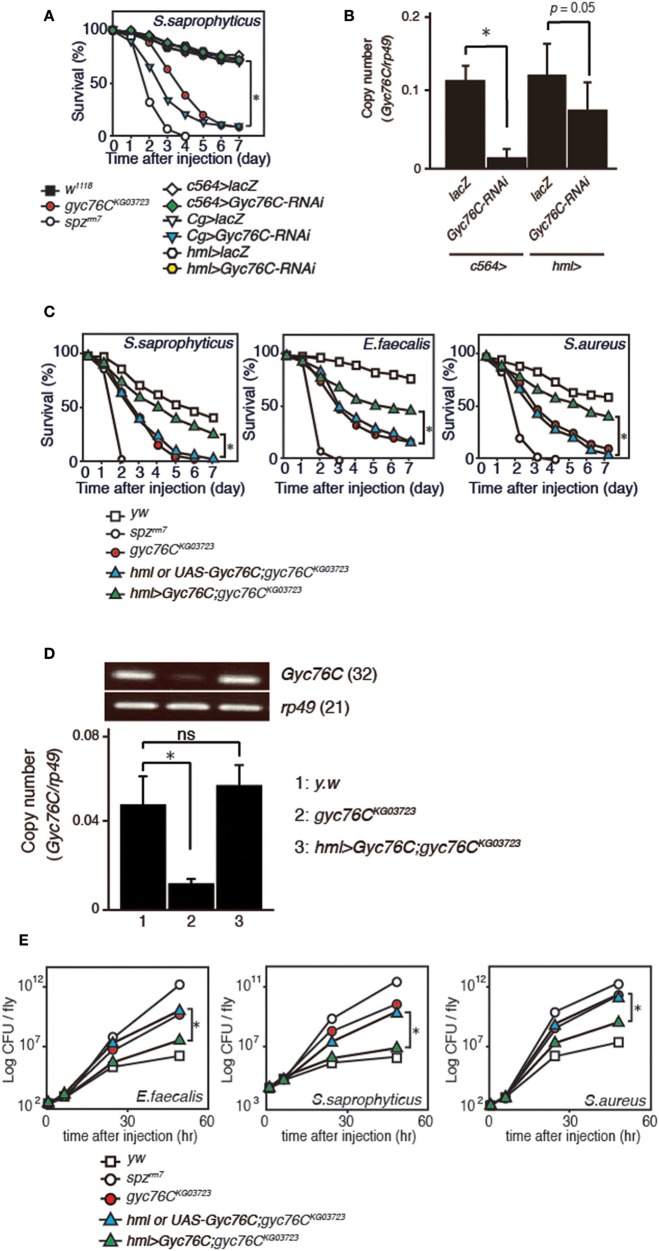
Requirement of *Gyc76C* expression in both the fat body and hemocytes for host survival against Gram-positive bacterial infections. **(A)** Effects of expression of *Gyc76C*-RNAi by three different GAL4 drivers, *Cg* (fat body and hemocyte)-, *c564* (fat body)-, and *hml* (hemocyte)-GAL4 drivers, on the survival rate against *S. saprophyticus* infection. **(B)** Effects of *Gyc76C* RNAi using two different GAL4 drivers (*c564*-, and *hml*-GAL4) on *Gyc76C* expression in flies. **(C)** Effects of hemocyte-specific expression of *Gyc76C* in *gyc76C*^*KG*03723^ flies by *hml*-GAL4 on the survival rate against *S. saprophyticus, E. faecalis*, and *S. aureus* infections. Siblings (*hml*-GAL4; *gyc76C*^*KG*03723^ or UAS-Gyc76C; *gyc76C*^*KG*03723^) were used as controls. **(D)** Semi-quantitative (upper) and quantitative (lower) RT-PCR analysis of the expression of *Gyc76C* in hemocytes isolated from *gyc76C*^*KG*03723^ larvae expressing *Gyc76C* by *hml*-GAL4. **(E)** Suppression of Gram-positive bacterial loads by hemocyte-specific expression of *Gyc76C* in *gyc76C*^*KG*03723^ flies. Differences in bacterial loads in *gyc76C*^*KG*03723^ and *gyc76C*^*KG*03723^ flies expressing *Gyc76C* in hemocytes by CFU assay are indicated. Data shown are the means of 6 independent experiments with over 30 flies of each genotype examined at the same time. **(A,C)** *P < 0.05, Log-rank test. Data shown are presented as means of at least three independent experiments. **(B,D,E)** *P < 0.05, ns: *P* > 0.1, Student's *t*-test. Error bars indicate standard deviation. Data are representative of the results of three independent experiments.

We previously identified Gyc76C as an immune receptor that is crucial for host survival against Gram-positive bacterial infections in *Drosophila* (Kanoh et al., under revision). *Gyc76C* is preferentially expressed in immune-related tissues such as the fat body, a major organ producing AMPs, hemocytes involved in cellular responses, and Malphigian (renal) tubules (Kanoh et al., under revision). To determine the tissue-specific requirement for *Gyc76C* in self-defense against Gram-positive bacteria, we investigated the effect of tissue-specific expression of RNA interference (RNAi) targeting *Gyc76C* using *Cg*-GAL4 (mainly in both the fat body and hemocytes), *c564*-GAL4 mainly in the fat body, but also in other tissues (31), and hemocyte-specific hemolectin (*hml*)*-*GAL4 (32) drivers in flies. Susceptibility to infection by *S. saprophyticus*, a Gram-positive bacteria, was induced by *Gyc76C* RNAi using *Cg*-GAL4 as previously reported (Kanoh et al., under revision), but not by *Gyc76C* RNAi using *c564*-GAL4 and *hml*-GAL4 (Figure 1A). *Gyc76C* expression in flies was partially reduced by *hml*-GAL4-mediated RNAi, but strongly reduced (90% reduction) by *c564*-GAL4-mediated RNAi (Figure 1B) similar to *Cg*-GAL4-mediated RNAi (Kanoh et al., under revision). These findings suggest that *Gyc76C* expression, mainly in both the fat body and hemocytes, is required for self-defense against Gram-positive bacteria. Demonstrating a role for *Gyc76C* in hemocytes in self-defense, hemocyte-specific expression of *Gyc76C* by *hml*-GAL4 in *gyc76C*^*KG*03723^ a hypomorphic mutant fly (33), partially rescued the phenotype susceptible to Gram-positive bacterial infections (*S. saprophyticus, E. faecalis*, and *S. aureus*; Figure 1C). *Gyc76C* expression in larval hemocytes was completely rescued by hemocyte-specific expression of *Gyc76C* induced by *hml*-GAL4 in the *gyc76C*^*KG*03723^ mutant (Figure 1D). Colony formation unit assay further demonstrated that while *Gyc76C*^*KG*03723^ mutant flies accumulated significant Gram-positive bacterial loads in their hemolymph as reported previously (Kanoh et al., under revision), hemocyte-specific expression of *Gyc76C* in *gyc76C*^*KG*03723^ flies conversely suppressed Gram-positive bacterial growth (*E. faecalis, S. saprophyticus*, and *S. aureus*) in the hemolymph (Figure 1E). Taken together, these results indicate a self-defense role of Gyc76C in hemocytes.

### Role of Gyc76C in Cellular Responses Against Bacterial Infections

**Figure 2:**
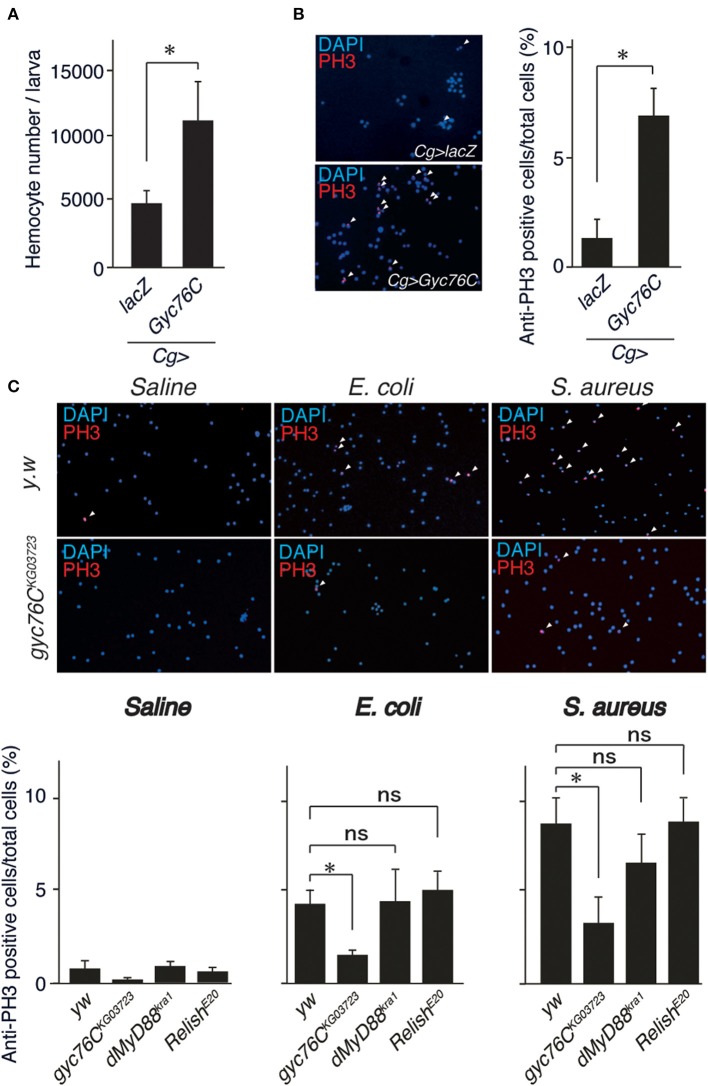
Role of Gyc76C in cellular responses against bacterial infections. **(A,B)** Total hemocyte number **(A)** and percentage of anti-PH3–positive cells **(B)** of *Gyc76C*-expressing larvae. Hemocyte nuclei were visualized by DAPI (blue); the proliferated hemocytes were stained with anti-PH3 antibody (red, arrowheads). *LacZ* was expressed using the same GAL4 drivers as used for the controls. **(C)** Bacterial infection-dependent hemocyte proliferation after ~3 h was monitored by anti-PH3 antibody staining with *yw* (control), *gyc76C*^*KG*03723^, *dMyD88*^*kra*1^, and *Relish*^*E*20^ mutant larvae. *P < 0.05, ns: *P* > 0.1, Student's *t*-test. Error bars indicate standard deviation. Data shown are representative of at least three independent experiments.

Because *Gyc76C* expression in hemocytes is necessary for self-defense, we investigated the role of *Gyc76C* in cellular responses against bacterial infections. The number of hemocytes in the hemolymph collected from larvae overexpressing *Gyc76C* by *Cg*-GAL4 was significantly increased compared with that of control larvae expressing *lacZ* (Figure 2A). Consistent with this finding, immunofluorescence analysis with an antibody specific for phosphorylated histone H3, a marker for entry into mitosis, revealed that *Gyc76C* overexpression by *Cg*-GAL4 significantly increased the number of proliferating hemocytes in the larvae compared with control larvae expressing *lacZ* (Figure 2B). Similar results were obtained in studies of bromodeoxyuridine incorporation into hemocytes (data not shown). Moreover, a similar increase in hemocyte proliferation was induced in larvae by infection with *E. coli*, a Gram-negative bacteria, and *S. aureus*, a Gram-positive bacteria, as well as by injection of control saline, and the hemocyte proliferation was reduced in *gyc76C*^*KG*03723^ (Figure 2C). Activation of the Toll pathway induces lamellocyte differentiation as well as hemocyte proliferation (3, 34, 35). On the basis of their morphology, however, lamellocyte differentiation was not induced by *Gyc76C* overexpression, which is consistent with reports that lamellocyte differentiation and hemocyte proliferation are independently controlled (17, 18). These findings together indicated that *Gyc76C* affects the basal level of hemocyte proliferation.

### Gyc76C Mediates Hemocyte Proliferation as a Cellular Response in a Distinct Way From the Humoral Response

**Figure 3:**
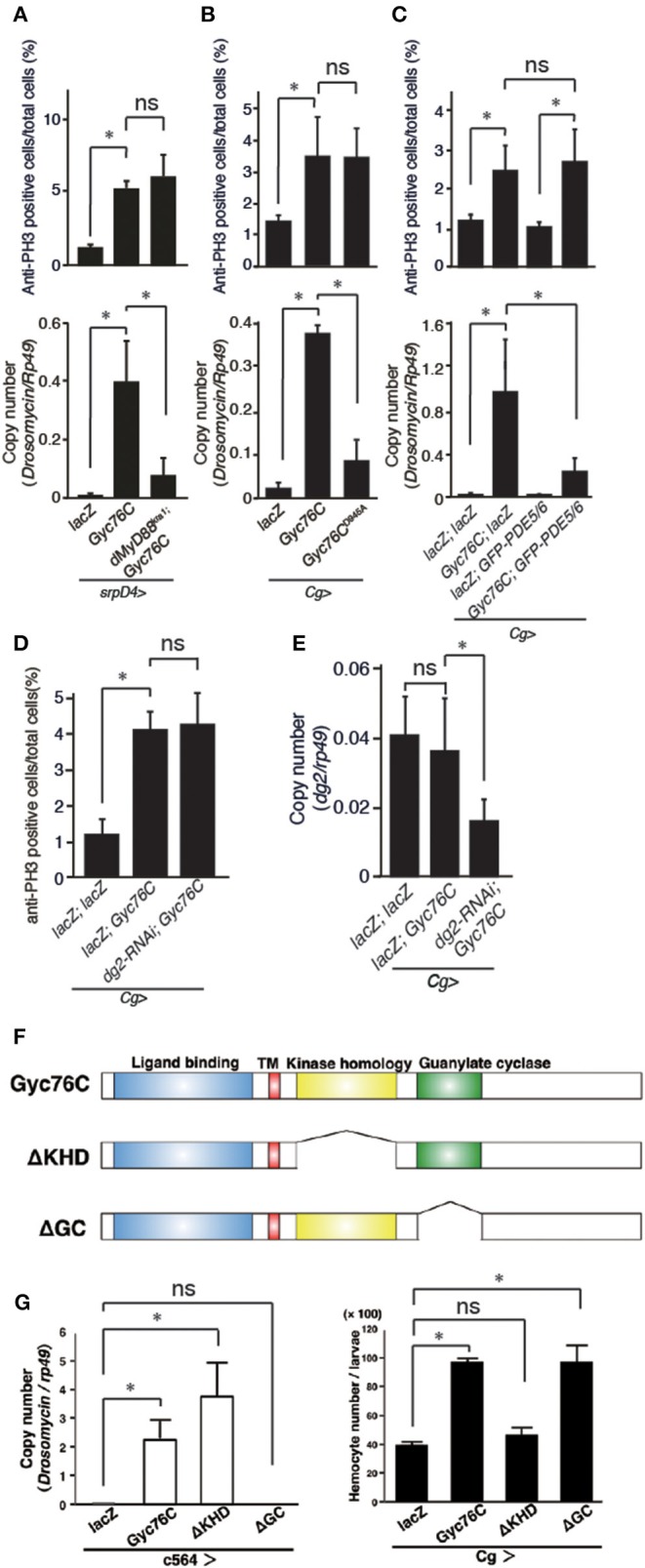
Gyc76C mediates hemocyte proliferation in a distinct way from the humoral response. **(A)** Effects of overexpression of *Gyc76C* in wild-type and *dMyD88*^*kra*1^ background larvae on hemocyte proliferation and *Drs* expression. **(B)** Effects of *Gyc76C*^*D*945*A*^ expression in larvae on hemocyte proliferation and *Drs* expression. **(C)** Effects of *PDE5/6* expression on *Gyc76C*-mediated hemocyte proliferation and *Drs* expression. *LacZ* expression by the same GAL4 driver was used as a control. *Drs* expression was measured in whole larvae. **(D)** Effects of expression of RNAi targeting *dg2* in larvae on *Gyc76C*-mediated hemocyte proliferation. *LacZ* was expressed using the same GAL4 drivers as used for the controls. **(E)** Effect of *dg2* RNAi induced by *Cg*-GAL4 on *dg2* expression in hemocytes. *LacZ* was expressed using the same GAL4 drivers as used for the controls. **(F)** Schematic representation of the domain structure of wild-type Gyc76C protein and deletion mutants used in this study. **(G)** Effects of expression of wild-type Gyc76C and Gyc76C mutants lacking the KHD (ΔKHD) and GC domains (ΔGC) in larvae on *Drs* expression and hemocyte number. *P < 0.05, ns: *P* > 0.1, Student's *t*-test. Error bars indicate standard deviation. Data shown are representative of at least three independent experiments.

The bacterial infection-dependent hemocyte proliferation in larvae was not affected in *dMyD88*^*kra*1^, a mutant of the dMyD88 adaptor protein in the Toll pathway, and *Relish*^*E*20^, a mutant of the Relish transcription factor of the imd pathway, suggesting that neither the Toll nor the imd pathway is involved in bacterial infection-dependent hemocyte proliferation (Figure 2C). Consistently, *Gyc76C*-mediated induction of *Drs* in larvae was suppressed by the *dMyD88*^*kra*1^ mutation as reported previously (Kanoh et al., under revision), whereas *Gyc76C*-mediated induction of hemocyte proliferation was not affected by the *dMyD88*^*kra*1^ mutation, indicating that Gyc76C mediates hemocyte proliferation in a *dMyD88*-independent manner (Figure 3A). Surprisingly, hemocyte proliferation was also induced by overexpression of the *Gyc76C*^*D*945*A*^ mutant, which produces low levels of cGMP and has low *Drs* expression in larvae (Kanoh et al., under revision), as well as by wild-type *Gyc76C* (Figure 3B). Moreover, as shown in Figure 3C, *Gyc76C*-mediated hemocyte proliferation was not affected by the expression of *PDE5/6*, which severely reduces both *Gyc76C*-mediated *Drs* induction and cGMP production in larvae (Kanoh et al., under revision). The *Gyc76C*-dependent induction of *Drs* is inhibited by the expression of RNAi targeting *dg2*, a gene of cGK, in the fat body driven by *c564*-GAL4 (Kanoh et al., under revision), whereas *Gyc76C*-dependent hemocyte proliferation was not affected by the expression of RNAi targeting *dg2* in the fat body and hemocytes driven by *Cg*-GAL4 in larvae (Figure 3D). Expression of *dg2* in larval hemocytes was reduced by *dg2* RNAi using *Cg*-GAL4 (Figure 3E). Gyc76C has an extracellular ligand-binding domain, a transmembrane domain, intracellular kinase homology, and guanylate cyclase domains, which show amino acid sequence similarity to rGCs, including mammalian rGCs (36) (Figure 3F). Expression of a Gyc76C mutant lacking the kinase homology domain (KHD) in larvae induced relatively higher *Drs* expression compared with wild-type Gyc76C, but a Gyc76C mutant lacking the guanylate cyclase (GC) domain failed to induce *Drs* expression (Figure 3G), consistent with a previous study demonstrating that deletion of the KHD led to an increase in the GC activity of Gyc76C in *Drosophila* S2 cells (37). Hemocyte proliferation was induced by the expression of a Gyc76C mutant lacking GC as well as by wild-type Gyc76C, but not by a Gyc76C mutant lacking the KHD in larvae (Figure 3G). These results indicate that Gyc76C mediates hemocyte proliferation in a cGMP-independent manner. Therefore, Gyc76C mediates humoral and cellular responses by distinct mechanism. The humoral response such as AMP induction is mediated by the producing cGMP and through cGK and dMyD88 (Kanoh et al., under revision), whereas a cellular response, hemocyte proliferation, is cGMP-independent.

### Gyc76C Mediates ModSP-Dependent Hemocyte Proliferation as Well as ModSP-Dependent *Drs* Expression

**Figure 4:**
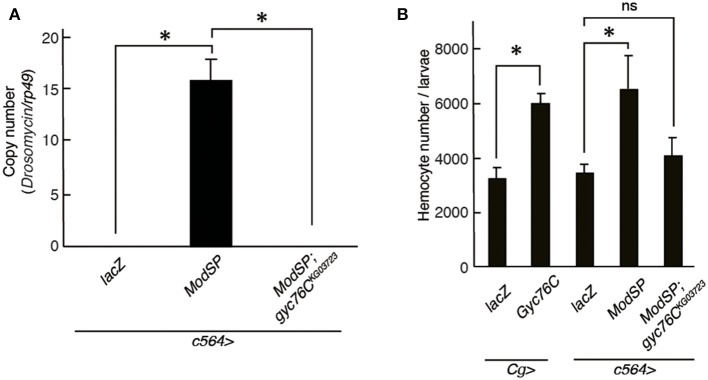
Gyc76C is required for the ModSP-dependent increase in hemocyte number as well as ModSP-dependent *Drs* expression. **(A)** Effects of *Gyc76C* mutation on the ModSP-dependent induction of *Drs* in larvae. **(B)** Gyc76C- and ModSP-dependent increase in the hemocyte number in larvae, and effects of *Gyc76C* mutation on the ModSP-dependent increase in the hemocyte number. Circulating hemocytes were collected from *Gyc76C*-overexpressing larvae by *Cg*-GAL4, *ModSP*-overexpressing larvae by *c564*-GAL4, *ModSP*-overexpressing *gyc76C*^*KG*03723^ mutant larvae by *c564*-GAL4, and *lacZ*-expressing larvae by *Cg*-GAL4 and by *c564*-GAL4 (control). *P < 0.05, ns: *P* > 0.1, Student's *t*-test. Error bars indicate standard deviation. Data shown are representative of at least three independent experiments.

*Drs* is induced by the overexpression of ModSP, an upstream regulator of the Toll receptor (12). As reported previously (Kanoh et al., under revision), the *Drs* induction by overexpression of *ModSP* in the fat body (*c564*-GAL4) was suppressed in *gyc76C*^*KG*03723^ mutant larvae (Figure 4A), indicating that the ModSP-dependent induction of *Drs* requires Gyc76C. Overexpression of *ModSP* in the fat body also increased the total number of hemocytes, the same as overexpression of *Gyc76C* in hemocytes and the fat body by *Cg*-GAL4 in larvae (Figure 4B). The ModSP-dependent increase in the hemocyte number was suppressed in *gyc76C*^*KG*03723^ mutants, indicating that the ModSP-dependent increase in the hemocyte number also requires Gyc76C (Figure 4B). Therefore, although the Gyc76C-mediated humoral and cellular responses are differentially regulated downstream of Gyc76C, both responses are triggered by *ModSP* overexpression.

### Gyc76C-Dependent Cellular Response Is Mediated by a Small GTPase, Ras85D

**Figure 5:**
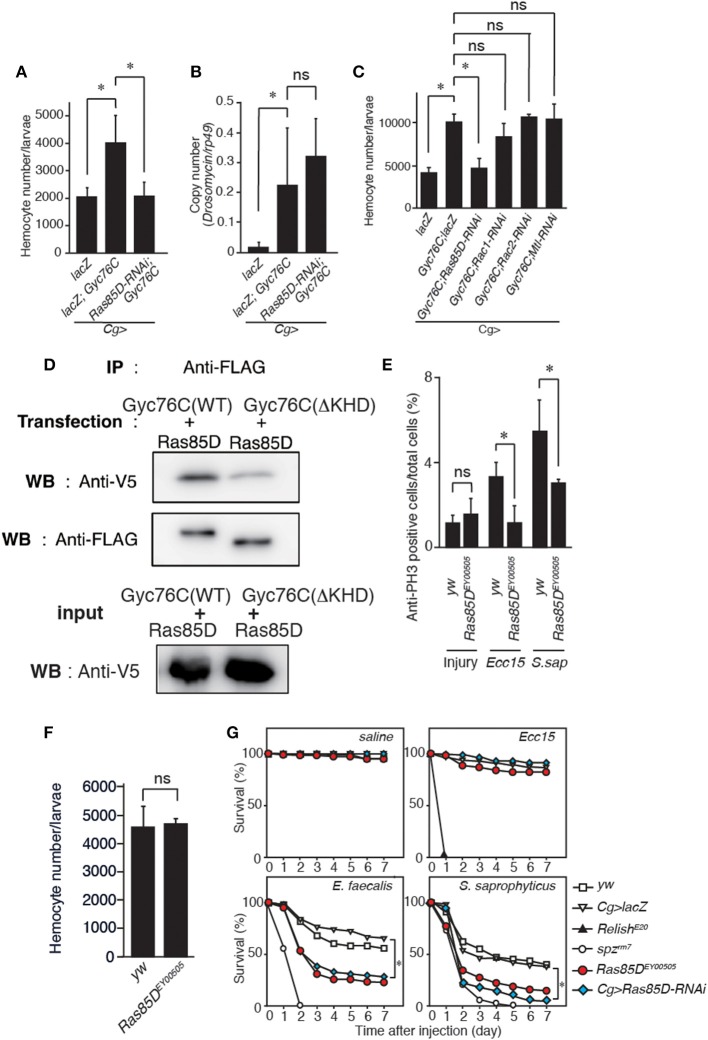
Gyc76C-dependent hemocyte proliferation is mediated by a small GTPase, Ras85D. **(A,B)** Effects of the expression of RNAi targeting *Ras85D* in larvae on *Gyc76C*-mediated hemocyte proliferation **(A)**, *Gyc76C*-mediated *Drs* induction **(B)**. *LacZ* was expressed using the same GAL4 drivers as used for the controls. **(C)** Effects of expression of RNAi targeting *Rac1, Rac2, and Mtl* in larvae on *Gyc76C*-mediated hemocyte proliferation. *LacZ* was expressed using the same GAL4 drivers as used for the controls. **(D)** Co-immunoprecipitation of Ras85D with wild-type (WT) Gyc76C or with Gyc76C mutants lacking the KHD (ΔKHD). FLAG-tagged wild-type Gyc76C or FLAG-tagged ΔKHD Gyc76C mutant was expressed with V5-tagged Ras85D in S2 cells. Immunoprecipitation (IP) was performed with anti-FLAG antibody, and then Western blotting (WB) was performed using anti-V5 and anti-FLAG antibodies, respectively. **(E)** Bacterial infection (*Ecc15, S. saprophyticus*)-dependent hemocyte proliferation was monitored by anti-PH3 antibody staining with *yw* (control), and *Ras85D*^*EY*00505^ mutant larvae. **(F)** The number of hemocytes in *Ras85D*^*EY*00505^ mutant larvae in the absence of infection. Circulating hemocytes were collected from *Ras85D*^*EY*00505^ mutant and control (*yw*) larvae. **(G)** *Ras85D* is required for host defense against Gram-positive bacterial infection. Survival rate of control (*yw, lacZ-*expressing flies), *Ras85D* RNAi using *Cg*-GAL4, *Ras85D*^*EY*00505^, *spz*^*rm*7^, and *Relish*^*E*20^ flies was tested after injecting saline (as a control), Gram-negative bacteria (*Ecc15*), or Gram-positive bacteria (*E. faecalis* and *S. saprophyticus*) at 28°C. **(A–F)** *P < 0.05, ns: *P* > 0.1, Student's *t*-test. Data shown are representative of at least three independent experiments. Error bars indicate standard deviation. **(G)** *P < 0.05, Log-rank test. Data shown are presented as means of at least three independent experiments.

**Figure 6:**
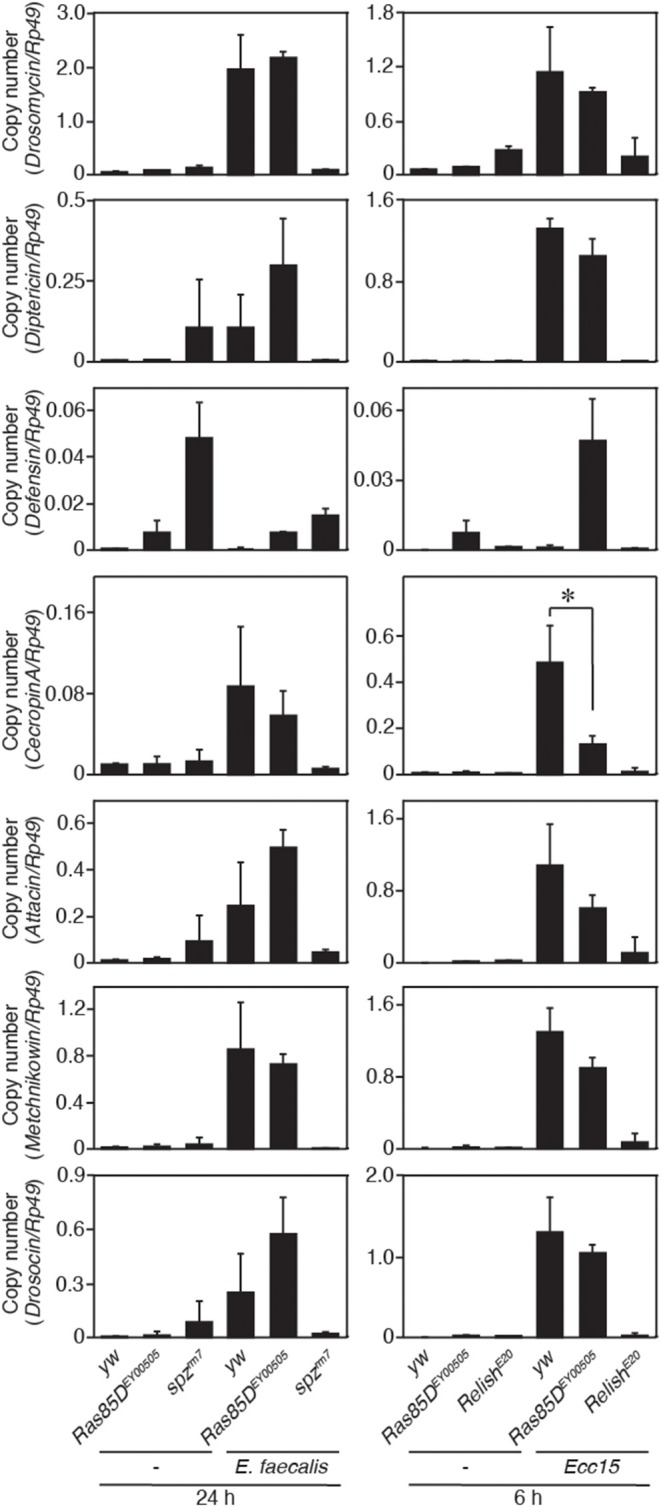
Expression of antimicrobial peptide genes in a *Ras85D* mutant after bacterial infection. Either 24 h after *E. faecalis* injection or 6 h after *Ecc15* injection, the expression of 7 distinct AMPs was measured with the P-element insertion mutant of *Ras85D, Ras85D*^*EY*00505^, *spz*^*rm*7^, and *Relish*^*E*20^, and *yw* flies (used as a control). Because Ras85D is reported to be involved in constitutive repression of the imd pathway (38), the values of uninfected flies are also presented. Data shown are the means of at least three independent experiments. *P < 0.05, Student's *t*-test. Error bars indicate standard deviation.

A small GTPase, Ras85D, is suggested to be involved in hemocyte proliferation (17). We investigated the effect of expressing RNAi targeting *Ras85D* and other small GTPase superfamily members, *Rac1, Rac2*, and Mig-2-like (*Mtl*), on *Gyc76C*-dependent hemocyte proliferation and *Gyc76C*-dependent induction of *Drs* in larvae. *Gyc76C*-dependent hemocyte proliferation was reduced by *Ras85D* RNAi using *Cg*-GAL4, whereas *Gyc76C*-dependent induction of *Drs* was not affected by *Ras85D* RNAi (Figures 5A,B). Expression of RNAi targeting *Rac1, Rac2*, and *Mtl* did not inhibit the *Gyc76C*-dependent hemocyte proliferation in larvae (Figure 5C). Consistent with the functional interactions of *Gyc76C* and *Ras85D*, co-immunoprecipitation results revealed that Ras85D forms a complex with wild-type Gyc76C in *Drosophila* S2 cells (Figure 5D). The Ras85D-complex formation was reduced in a Gyc76C mutant lacking the KHD that does not induce hemocyte proliferation (Figures 5D, 3G). Moreover, infection-dependent hemocyte proliferation in larvae in response to *S. saprophyticus* and *Ecc15*, a Gram-negative bacteria, was reduced by a *Ras85D* mutation, *Ras85D*^*EY*00505^, caused by a P-element insertion in the 5′-untranslated region of *Ras85D* (Figure 5E), whereas in the absence of infection, the number of hemocytes was not affected in *Ras85D*^*EY*00505^ mutant larvae (Figure 5F). Therefore, Ras85D mediates hemocyte proliferation by Gyc76C in response to bacterial infections as a cellular response. *Cg*-GAL4-driven *Ras85D*-RNAi flies and *Ras85D*^*EY*00505^ flies were susceptible to Gram-positive bacterial infections (*E. faecalis* and *S. saprophyticus*), but not to *Ecc15* infection (Figure 5G). The response of *Ras85D*-RNAi flies to *Ecc15* infection was consistent with a previous report (38). AMP induction after *E. faecalis* and *Ecc15* infections was not reduced in *Ras85D*^*EY*00505^ compared with control flies (*yw*), except for *CecropinA1* against *Ecc15* infection (Figure 6). These findings suggest that the Ras85D plays an important role in the cellular innate immune response against Gram-positive bacterial infection. We cannot, however, exclude the possibility of a potential contribution of a humoral response, as observed by the dysregulated antimicrobial expression pattern in Ras85D mutant flies.

## Discussion

We previously reported that the Gyc76C mediates humoral response by a membrane-localized cGK, DG2 through downstream components of the Toll receptor via dMyD88 (Kanoh et al., under revision). In this study, we provide new evidence that the Gyc76C is also involved in cellular response. Further mechanistic analyses indicate that this Gyc76C-mediated cellular response is executed through a small GTPase, Ras85D, and importantly, this response is in cGMP-independent manner. The Gyc76C-mediated cellular responses confer host survival against Gram-positive bacterial infections, like the Gyc76C-mediated humoral responses. Similar to Ras85D, Gyc76C is involved in hemocyte proliferation in response to Gram-negative bacteria, but neither Gyc76C nor Ras85D is crucial for host survival against Gram-negative bacterial infections, suggesting that Gyc76C-mediated hemocyte proliferation does not confer host survival against Gram-negative bacterial infections. Gyc76C is not involved in the imd pathway-dependent AMP induction in response to Gram-negative bacterial infections (Kanoh et al., under revision). In comparison with AMP induction by the Toll pathway in response to Gram-positive bacterial infections, AMPs are rapidly induced by the imd pathway in response to Gram-negative bacterial infections in flies (39). Because of the rapid induction of AMPs by activation of the imd pathway, Gyc76C-mediated hemocyte proliferation might not be required for host survival against Gram-negative bacterial infections.

We demonstrated that both the Gyc76C-mediated humoral and cellular responses are triggered by the overexpression of *ModSP*. Although the ligand of Gyc76C that induces the Gyc76C-mediated humoral response in response to Gram-positive bacteria has not yet been identified (Kanoh et al., under revision), it is possible that the ligand produced by infection activates Gyc76C to induce both the humoral and cellular immune responses and thus coordinates them to eliminate the pathogens. Identification and characterization of the Gyc76C ligand is necessary to elucidate the coordination mechanisms of the humoral and cellular immune responses in *Drosophila*.

rGCs have two conserved intracellular domains, kinase homology and guanylate cyclase domains (36). The KHD regulates the activity of the associated GC domain (40). Deletion of the KHD of Gyc76C leads to increased GC activity in *Drosophila* S2 cells, indicating that the KHD of Gyc76C is also involved in regulating GC activity (35). In this report, we demonstrated that a Gyc76C mutant with deletion of the KHD induced *Drs* expression, but a Gyc76C mutant with deletion of the GC domain failed to induce *Drs* expression. Conversely, a Gyc76C mutant without the GC domain induced hemocyte proliferation, but a Gyc76C mutant without the KHD failed to induce hemocyte proliferation. These findings indicate that the KHD of Gyc76C has an independent role in regulating GC activity. Consistent with these analyses, co-immunoprecipitation analysis suggests that the KHD of Gyc76C is involved in the association with Ras85D that is required for Gyc76-dependent hemocyte proliferation. The KHD of Gyc76C may be involved in forming a signaling platform with other factors such as Ras85D. Additional studies are needed to clarify how the two independent functions of Gyc76C are regulated through the two functional domains of the receptor.

## Data Availability Statement

All datasets generated for this study are included in the article/supplementary material.

## Author Contributions

SI and AG performed hemocyte proliferation analyses. HS performed Ras85D analyses with help of HK and TK. TO performed ModSP analyses with help of NF and TY. YO promoted this study. S-AD and JD designed the cGMP studies. SK provided overall coordination with respect to conception, design, and supervision of the study, and wrote the manuscript with comments from co-authors. SI, HS, and AG contributed equally to the study. All authors discussed the results.

### Conflict of Interest

The authors declare that the research was conducted in the absence of any commercial or financial relationships that could be construed as a potential conflict of interest.
